# Effects of Salt Stress on Plant Growth, Antioxidant Capacity, Glandular Trichome Density, and Volatile Exudates of *Schizonepeta tenuifolia* Briq.

**DOI:** 10.3390/ijms19010252

**Published:** 2018-01-15

**Authors:** Ying Zhou, Nanyu Tang, Lijin Huang, Yongjuan Zhao, Xiaoqing Tang, Kangcai Wang

**Affiliations:** College of Horticulture, Nanjing Agricultural University, Nanjing 210095, China; 2016104130@njau.edu.cn (Y.Z.); 14415225@njau.edu.cn (N.T.); 14415226@njau.edu.cn (L.H.); 14415222@njau.edu.cn (Y.Z.)

**Keywords:** salt stress, *Schizonepeta tenuifolia* Briq., growth, antioxidant activities, glandular trichome density, volatile exudates

## Abstract

Salinity is a major abiotic factor affecting plant growth and secondary metabolism. However, no information is available about its effects on *Schizonepeta tenuifolia* Briq., a traditional Chinese herb. Here, we investigated the changes of plant growth, antioxidant capacity, glandular trichome density, and volatile exudates of *S. tenuifolia* exposed to salt stress (0, 25, 50, 75, 100 mM NaCl). Results showed that its dry biomass was reduced by salt treatments except 25 mM NaCl. Contents of antioxidants, including phenolics and flavonoids, increased at low (25 mM) or moderate (50 mM) levels, but declined at severe (75 and 100 mM) levels. On leaf surfaces, big peltate and small capitate glandular trichomes (GTs) were found. Salt treatments, especially at moderate and severe concentrations, enhanced the density of total GTs on both leaf sides. The most abundant compound in GT volatile exudates was pulegone. Under salinity, relative contents of this component and other monoterpenes decreased significantly; biosynthesis and accumulation of esters were enhanced, particularly sulfurous acid,2-ethylhexyl hexyl ester, which became the second major compound as salinity increased. In conclusion, salt stress significantly influenced the growth and secondary metabolism of *S. tenuifolia*, enabling us to study the changes of its pharmacological activities.

## 1. Introduction

*Schizonepeta tenuifolia* Briq. (Jingjie in Chinese) is an annual plant belonging to the Lamiaceae family. Now, it is cultivated for medicinal and culinary purposes, namely it is used as an ingredient in herbal medicines, beneficial teas, food recipes, sauces, and beverages. In China, Japan, and Korea, dried aerial parts of *S. tenuifolia* have long been popularly used as a traditional medicinal herb to treat headaches, colds, fevers, sore throats, allergic dermatitis, eczema, and psoriasis [1,2]. Modern pharmacological studies have shown that its methanolic or aqueous extracts have antioxidant, anti-inflammatory, antipruritic, and antiviral activities [3,4,5]. Like many plants in the Lamiaceae family, this medicinal and aromatic plant also produces essential oil (EO), which is considered the major material basis of its biological effects, and exhibits anti-inflammatory [6] and insecticidal [7,8] activity. Main components in EO distilled from *S. tenuifolia* aerial parts are monoterpenes including pulegone, d-limonene and menthone [1]. Pulegone, with a pleasant and refreshing odor, is commercially used in flavoring agents, perfumery, aromatherapy, and diverse pharmaceuticals [9]. In the Chinese Pharmacopoeia [10], pulegone was selected as the marker compound for quality control of schizonepetae herba. D-limonene, one of the most common terpenes in nature, is a principal component in several citrus oils (orange, lemon, grapefruit, mandarin, and lime). It is listed in the Code of Federal Regulation of the United States of America (USA) as generally recognized as safe for a flavor and fragrance additive in foods, perfumes, beverages, soaps, and chewing gum. Clinically, d-limonene is used for dissolving cholesterol-containing gallstones and relief of heartburn [11]. As for menthone, it is used in household products, components of artificial volatile oils, and tooth-brushing powder [12]. The EO is medicinally important since it is one of the raw materials of many Chinese Traditional Patent Medicines [13]. In the plant kingdom, there are specialized tissues and cell types responsible for synthesizing and accumulating secondary metabolites, such as secretory cavities, resin ducts, laticifers, glandular trichomes (GTs), and gum ducts [14,15]. While in the Lamiaceae species, the glandular trichome (GT) of the epidermal structure is the site that serves for the biosynthesis, secretion, and accumulation of EO [16]. Due to its location on the plant surface and the hydrophobic property of metabolites synthesized by GT, GT exudates can be recovered by simple organic solvent washes [17]. This accessibility has, therefore, made it possible for us to study the metabolic profiling of GTs, in addition to extracting essential oil by steam distillation.

Salinity is one of the major abiotic factors reducing global crop yield; it affects nearly 20% of the cultivated lands around the world and about 50% of all irrigated lands [18,19]. In China, there are about 34.6 million hectares of salinized lands. Among the major and vital groups of crops, the medicinal plants, which exert an important role in human disease prevention and treatment [20], are also being threatened by this constraint. It is well established that secondary metabolites in medicinal plants are involved in the treatment of human diseases and health disorders [21]. However, their accumulation is strongly dependent on growing conditions [22]. Among the secondary metabolites, polyphenolic compounds with strong antioxidant activities are abundant in the Lamiaceae plants [23]. Under salinity and other biotic/abiotic stresses, their synthesis and accumulation are generally vitalized [24]. Thus, it has been suggested that plants stressed by salinity might have the potential to be sources of polyphenols [25].

*S. tenuifolia* is able to grow in most regions of China but is mainly cultivated commercially in northeastern and northern China. In the past few years, these areas have been confronted with soil secondary salinization due to inappropriate fertilizer and irrigation management. Moreover, cultivation of *S. tenuifolia* is also being conducted on improved coastal saline soils. Thus, research concerning the impacts caused by salinity on the growth and secondary metabolism of *S. tenuifolia* is needed. In this study, we aimed to investigate the changes of plant growth and antioxidant capacity of *S. tenuifolia* in response to salt stress. Furthermore, GTs are specialized hairs found on plant aerial parts and are responsible for large portions of the plant’s secondary chemistry [26]. The occurrence of GTs in *S. tenuifolia* has compelled us to evaluate how salt stress affected their density and composition of volatile exudates. This study will deepen our understanding of aspects of secondary metabolic changes induced by salt stress in the traditional Chinese herb, *S. tenuifolia*.

## 2. Results

### 2.1. Plant Growth

**Figure 1:**
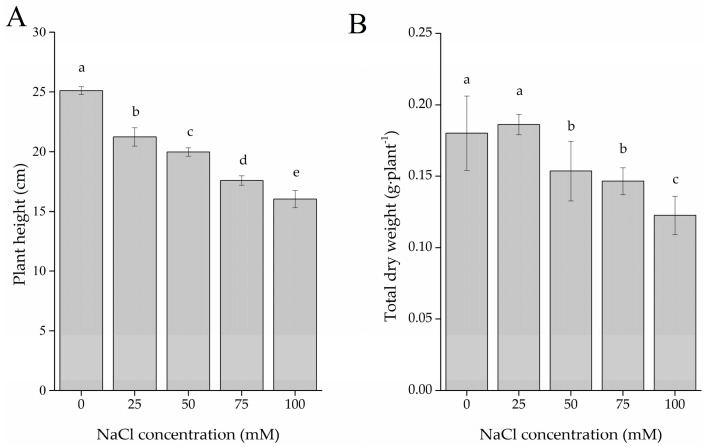
Plant growth of *S. tenuifolia* influenced by salt stress. (**A**) Plant height and (**B**) total dry weight of plants treated by five different NaCl concentrations. Data are expressed as the mean ± SD. Bars with different letters are significantly different at *p* < 0.05 (*n* = 10).

In Figure 1A, a substantial and obvious decrease in *S. tenuifolia* plant height was observed at 25, 50, 75, and 100 mM NaCl by 15.41%, 20.43%, 29.99%, and 36.13%, respectively, compared with the control. Total dry weight of plants exhibited a slight rise at 25 mM NaCl but reduced significantly by 14.44%, 18.33%, and 31.6% at 50, 75, and 100 mM NaCl, respectively, compared with the control.

### 2.2. Total Phenolic and Flavonoid Content (TPC and TFC)

As shown in Table 1, the total phenolic content (TPC) of *S. tenuifolia* leaf was measured as 13.24 mg GAE/g DW (mg of gallic acid equivalents on a basis of dry weight) in the control. At 25 mM NaCl, a significant increase in TPC was observed, which was 1.4 times higher than that of the control. However, higher concentrations of salinity caused a diminution in TPC. The TPC value at 50 and 75 mM NaCl was 14.56 and 12.37 mg GAE/g DW, respectively, which was deemed to be at the same level as the control. At 100 mM NaCl, the obvious decrease was of about 44% as compared to the control plants. Similar but not the same, the accumulation of flavonoids was enhanced by low (25 mM NaCl) to moderate levels (50 mM NaCl) of salinity but was inhibited by severe levels (75 mM, 100 mM NaCl).

### 2.3. Antioxidant Capacity Evaluation

The antioxidant capacity of *S. tenuifolia* leaf methanol extracts was determined by DPPH (2,2-diphenyl-1-picrylhydrazyl) and ABTS^·+^ (2,2′-azinobis-(3-ethylbenzothiazoline-6-sulfonic acid)) radical scavenging assay (Table 1). In the DPPH system, the antioxidant capacity of leaf extracts in control plants was 40.17%. In response to salinity, it was found to be the highest (62.14%) in plants treated by 25 mM NaCl, then declined significantly at 50 mM (44.79%). Significantly, leaf extracts of plants showed a much weaker antioxidant capacity with increasing NaCl concentrations to 75 and 100 mM, reducing by 26% and 54%, respectively, compared to the control. A similar trend was detected in ABTS^·+^ radical scavenging capacity, except that the results were insignificant at 0 and 50 mM NaCl.

### 2.4. Glandular Trichome Morphology and Density

**Figure 2:**
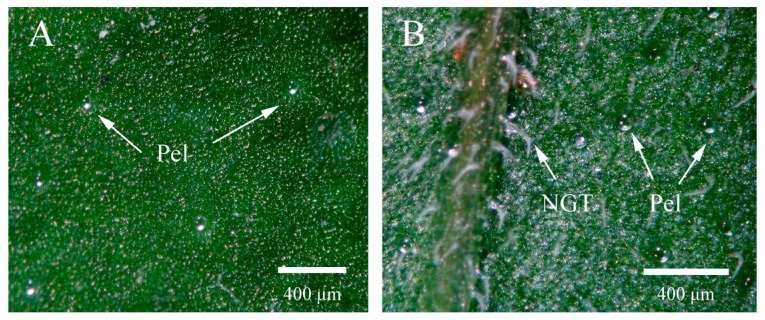
Stereomicroscope micrographs showing both sides of *S. tenuifolia* leaf. (**A**) Adaxial leaf surface with glandular peltate trichomes (Pels); (**B**) Abaxial leaf surface with non-glandular trichomes (NGTs) and glandular peltate trichome (Pels).

**Figure 3:**
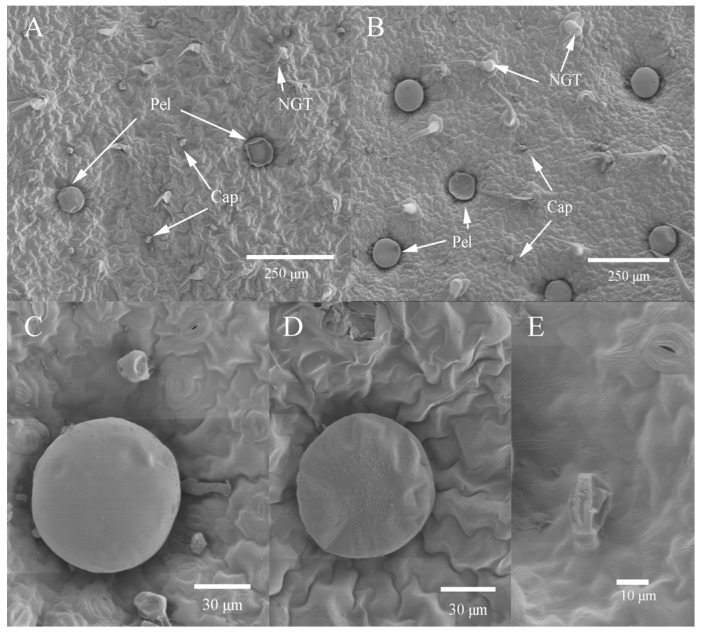
SEM (scanning electron microscope) micrographs showing the distribution and types of *S. tenuifolia* trichomes. (**A**) Adaxial leaf surface exhibiting three types of trichomes: non-glandular trichome (NGT), peltate glandular trichome (Pel) and capitate glandular trichome (Cap); (**B**) Abaxial leaf surface; (**C**) Peltate and capitate glandular trichomes on the leaf abaxial side; (**D**) Mature peltate glandular trichome; (**E**) Wrinkled capitate glandular trichome.

The indumentum of *S. tenuifolia* includes glandular trichomes (GTs) and non-glandular trichomes (NGTs) scattered on leaf surfaces (Figure 2 and Figure 3A,B). Considering the secreting potential, only the former type will be discussed here. The GTs are of two morphological types: peltate and capitate. Sunken in epidermal depressions, the peltate glandular trichomes (Pels) had a large spherical secretory head, 76 (±5) μm in diameter (Figure 3C,D). The capitate glandular trichomes (Cap) are much smaller and frequently appear shriveled and collapsed (Figure 3E).

**Figure 4:**
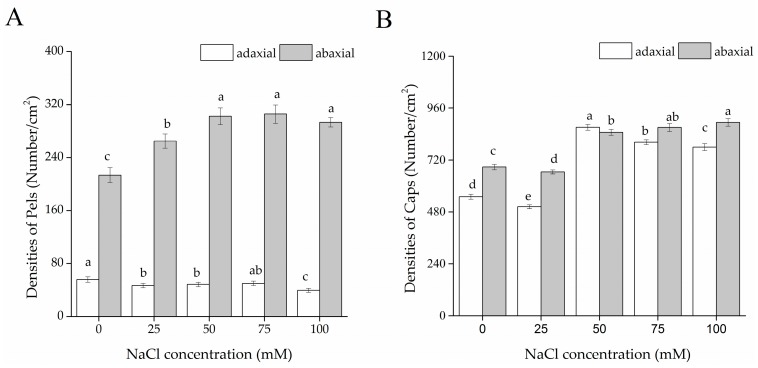
Effects of salt stress on the densities of glandular tricomes on leaves of *S. tenuifolia*. (**A**) Densities of Pels on both leaf surfaces; (**B**) Densities of Caps on both leaf surfaces. Data are expressed as the means ± SD. Bars filled in the same color with different letters are significantly different at *p* < 0.05 (*n* = 15).

As shown in Figure 4, the Pels were less abundant on the adaxial than the abaxial side. The Caps displayed the same distribution except with treatment of 50 mM NaCl. On the leaf abaxial sides, the densities of glandular trichomes of both types notably increased under moderate to severe NaCl concentrations compared to that of the control. The Pels on the leaf adaxial side decreased with increasing NaCl concentration and eventually reduced sharply at 100 mM NaCl; the Caps on the adaxial side of salt treatments, except those exposed to 25 mM NaCl, were more abundant than those of the control, and the highest density was observed at 50 mM NaCl. Nevertheless, the density of total GTs of both leaf sides was noticeably enhanced by salt stress, especially at moderate to severe salinities.

### 2.5. Constituents of Glandular Trichome Volatile Exudates in S. tenuifolia

Compositions of GT volatile exudates of *S. tenuifolia* and their relative percentage (%) at different NaCl levels are shown in Table 2. Fourteen compounds were identified in the control, accounting for 99.31% of the total GT volatile exudates. Pulegone (78.66%) was the major component; other notable components were menthone (5.48%), 2-cyclohexen-1-ol,2-methyl-5-(1-methylethenyl) (3.48%), 1,6-cyclodecadiene,1-methyl-5-methylene-8-(1-methylethyl) (3.02%), β-caryophyllene (2.27%), heptane,2,2,4,6,6-pentamethyl (2.44%), and d-limonene (1.54%). When exposed to salt stress, the relative proportions of these compounds were affected significantly. Compared with the control, the application of NaCl decreased the amounts of pulegone prominently by 19%, 19%, 39%, and 61% at 25, 50, 75, and 100 mM, respectively, but still remained the most abundant compound. Relative contents of 1,6-cyclodecadiene,1-methyl-5-methylene-8-(1-methylethyl) and β-caryophyllene diminished at 25 and 50 mM NaCl compared with the control; then, both compounds disappeared at 75 and 100 mM NaCl. The application of 25 mM NaCl did not notably change the d-limonene content with respect to the control. However, the level of 50 mM NaCl caused a significantly negative effect on its content and eventually the compound was not detected at levels of 75 and 100 mM. Menthone percentage at 25 and 50 mM NaCl was 2.14 and 1.97 times higher than that of the control, respectively, while it reduced significantly at 75 and 100 mM, especially the highest concentration. In contrast, NaCl stress enhanced the amounts of heptane,2,2,4,6,6-pentamethyl, 3-hexanone,2,2-dimethyl, 3-hexanone,2,5-dimethyl, and anisic acid,tridec-2-ynyl ester. It is notable that 2-cyclohexen-1-ol,2-methyl-5-(1-methylethenyl) disappeared when the plants were challenged by salt stress; while salt stress induced the biosynthesis of a new compound, sulfurous acid,2-ethylhexyl hexyl ester. As salinity increased, the amount of sulfurous acid,2-ethylhexyl hexyl ester increased remarkably and eventually became the second most abundant compound. In conclusion, GT volatile exudates in *S. tenuifolia* were rich in monoterpenes but they were affected negatively by salt stress. The biosynthesis of sesquiterpene, a minor class, was inhibited and then disappeared at 75 and 100 mM NaCl. On the contrary, other chemical classes, especially the esters, revealed a rising trend and gradually became the dominant chemical class in salt-treated plants.

**Figure 5:**
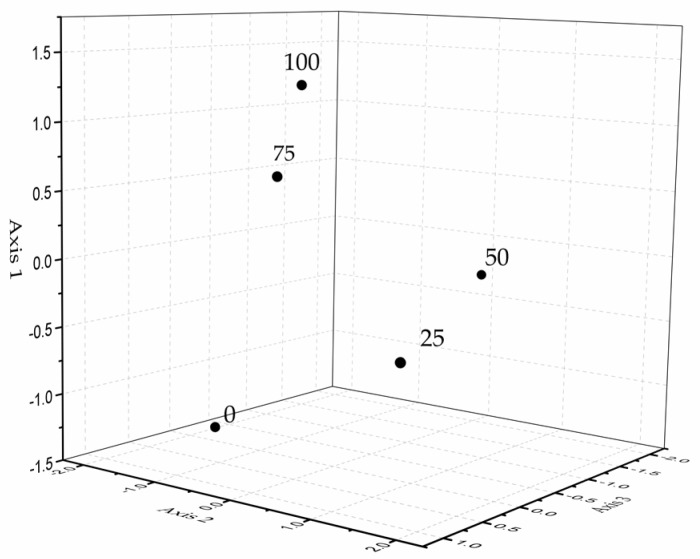
Relative position of five salt treatments (0, 25, 50, 75, 100 mM NaCl) in the space defined by three principle components.

Principle component analysis (PCA) was conducted to determine the relationship between different salt concentrations based on GT volatile exudate compositions (Figure 5). A clear distinction was revealed in the box plot of scores in three principle components. Firstly, the control (0 mM NaCl) was highly deviated from salt treatments. Secondly, salt levels of 25 and 50 mM NaCl formed a group. Higher levels of 75 and 100 mM NaCl formed the third group.

## 3. Discussion

In this investigation, *S. tenuifolia* growth was inhibited by salt stress in terms of plant height. Considering that no negative effect was found on the accumulation of dry matter by the application of 25 mM NaCl, it appears that *S. tenuifolia* can be tolerant to mild salinity, while plant height depressed remarkably at higher concentrations. Several authors have reported the reduction of biomass induced by salt stress in other medicinal and aromatic plants [27,28,29]. This inhibitory effect was probably due to the impact of salt on the stomata and photosynthesis process, as intercellular CO_2_ concentration was reduced and photosynthetic enzymes, chlorophylls, and carotenoids were disturbed, respectively [23].

The results showed that accumulation of the phenolics was induced under mild salinity (25 mM) but depressed under severe treatments (75 and 100 mM). It is well established that abiotic stresses including salinity cause oxidative damages, mainly by generating excess ROS (reactive oxygen species), which can attack lipids, proteins, DNA, and carbohydrates. The ROS is comprised of both non-radical (molecular) (^1^O_2_ and H_2_O_2_) and free radical forms (OH•, O_2_•^−^, RO• and HO_2_•) [30]. In order to scavenge or detoxify ROS, antioxidants such as phenolic compounds are produced by plants [31], and this is why the biosynthesis of such compounds is generally stimulated in salt-exposed plants [32]. However, the accumulation of phenolics under salinity conditions may vary in different plant species. In previous reports, the volume of phenolic compounds increased in buckwheat sprout [33], *Salvia mirzayanii* [34], and *Carthamus tinctorius* flowers [35] under NaCl stress, but failed to accumulate in coriander fruits [25] and baby Romaine lettuce [36] in response to NaCl treatments. A relative tolerance of *S. tenuifolia* to 25 mM NaCl was indicated as phenolic contents increased at that level. At 75 and 100 mM NaCl, scavenging ROS is inefficient by virtue of the imbalance between antioxidants formation and ROS. Oxidative damage such as leaf chlorosis and necrosis was consequently caused. Besides, increasing evidence suggested that flavonoids in higher plants have the potential to serve as antioxidants in response to environmental stresses including salinity [37,38,39,40,41]. It has been hypothesized that the biosynthesis of flavonoids, especially flavonol, can be stimulated by the changes of the cellular redox homeostasis [42] as MYB (myeloblastosis) transcription factors, which are involved in flavonol biosynthesis, are regulated by varied cellular redox potentials [43,44,45]. Changes in the overall results of DPPH and ABTS tests were similar to those observed in TPC. These antioxidant capacities may be directly linked with the amounts of phenolic compounds because of their free radical scavenging capacities [46,47]. Furthermore, in buckwheat sprout [33], maize [48], and some Chinese medicinal plants [49], total phenolic amounts were significantly correlated with antioxidant capacity.

Peltate (Pel) and capitate (Cap) glandular tricomes were observed on both leaf surfaces; this was considered as a common arrangement in the Lamiaceae family [50]. Compared to the Pels, the Caps were much smaller and most were shriveled and collapsed, demonstrating an apparently relatively short-lived property [51]. Regardless of the leaf side of *S. tenuifolia*, the Caps are distributed densely and the Pels scattered among them, as described in *Menta pulegium* [52,53]. Generally, Pels and Caps were more abundant on the abaxial than the adaxial side. When exposed to salt stress, the density of total GTs of both leaf sides increased, especially at moderate to severe salinities. As to data, there is very little available data on the effect of salt on the density of GT on leaf surfaces, particularly in Lamiaceae plants. Only one previous study showed that application of 50 mM NaCl improved the density of GTs on leaves of *M. pulegium* [52]. The increase of GT density can be explained by the fact that salt-treated (50–100 mM NaCl) plants exhibited less leaf area compared to that of the control, considering the fact that trichomes or oil glands are genetically fixed [54,55]. Could the increase of GT density in *S. tenuifolia* under salt stress improve EO yield? This is the next step of our research.

In this experiment, the GT volatile exudates of *S. tenuifolia* treated by salt stress were extracted by methylene chloride (CH_2_Cl_2_). According to Wagner et al. [56], washing leaf surfaces with CH_2_Cl_2_ can quickly and completely remove large quantities of GT exudates. Moreover, rapid rinsing with CH_2_Cl_2_ does not visibly penetrate the epidermis, i.e., it appears not to extract internal leaf metabolites. It is, therefore, convenient and efficient to study the impacts of salt stress on the aspect of GT secondary metabolism in this method as compared to steam distillation. 

As previously mentioned in *S. tenuifolia* EO, common components including pulegone (the most abundant compound), menthone, and d-limonene in GT volatile exudates were detected. However, relative percentages of these and other compounds underwent significant changes under salt stress. We found that proportions of pulegone declined with increasing salt concentrations. Pulegone was derived from d-limonene by a series of steps; it serves as a precursor for the production of methone by pulegone reductase (PR) [57]. In addition to the decrement of d-limonene, the decrease of pulegone could be due to the salt-induced negative impacts on the activity of related biosynthesis enzymes responsible for converting d-limonene to pulegone. The production of menthone was stimulated at mild and moderate salinity while it was inhibited by the most severe salt treatment; this may be related to the changes of PR activity under salt treatment. Terpenes including 1,6-cyclodecadiene,1-methyl-5-methylene-8-(1-methylethyl), β-caryophyllene, and d-limonene were not even detected at 75 and 100 mM. The amount of other compounds such as heptane,2,2,4,6,6-pentamethyl, 3-hexanone,2,2-dimethyl, 3-hexanone,2,5-dimethyl, and anisic acid,tridec-2-ynyl ester were increased by salinity. Moreover, a new compound, sulfurous acid,2-ethylhexyl hexyl ester, appeared under salt stress and eventually became the second most abundant compound, suggesting that salt stress has significant effects on the ester metabolic pathways of *S. tenuifolia*. We found that those variations were based on relative proportions of some constituents, as well as the absence of particular components or the presence of new ones. Consequently, results from the PCA indicated that the GT volatile exudates in *S. tenuifolia* are sensitive to salt stress, and this influence was dose-dependent. Since the essential oil is biosynthesized and stored in GT [58,59], the above phenomena alert us to investigate how salt stress influences the essential oil yield and components of *S. tenuifolia* and its medicinal and edible properties.

## 4. Materials and Methods

### 4.1. Plant Material and Salt Treatments

We performed this experiment in a greenhouse of the College of Horticulture, Nanjing Agricultural University, Nanjing, China. Plants were cultivated under a natural light condition with 30 °C day maximum and 15 °C night minimum, and 60–80% air humidity. *Schizonepeta tenuifolia* Briq. seeds were bought from Xincheng Chinese Herbal Medicine Industry (Anguo, China). In March, 2017, seeds were sown in trays containing a compost of humus, vermiculite, and perlite (1:2:1) and irrigated with distilled water to keep moist. About 8 days later, seeds were germinated and quarter-strength modified Hoagland’s solution [60] was used for irrigation. Thirty-seven days later (establishment phase), homogenous plants with a height of nearly 15 cm were transplanted into plastic pots filled with pure quartz sand. Two plants were cultivated in each pot and irrigated with 200 mL half-strength modified Hoagland’s solution every second day. Six days later, the plants were divided into five groups and salt treatments were initiated. A total of 300 mL of the above nutrient solution supplemented with 0, 25, 50, 75, or 100 mM NaCl was applied every day. To prevent osmotic shock, salt concentrations increased gradually with 25 mM NaCl every other day until the designated concentration was reached. The experimental design was completely randomized with 70 individuals for each treatment. All plants were harvested after 12 days since salt stress symptoms (leaf chlorosis and necrosis) occurred, especially in those treated with 75 and 100 mM NaCl.

### 4.2. Chemicals

Folin-Ciocalteu reagent, gallic acid (GA) and rutin were purchased from Yuanye Bio-Technology Co. (Shanghai, China). 2,2′-azinobis-(3-ethylbenzothiazoline-6-sulfonic acid) (ABTS) and 2,2-diphenyl-1-picrylhydrazyl (DPPH) were purchased from TCI Development Co. (Shanghai, China). All other reagents were purchased from Xilong Scientific Co. (Guangzhou, China).

### 4.3. Plant Growth Parameters

Ten individual plants for each treatment were collected to record the plant height and fresh weight. Their dry weight was measured after being oven-dried at 50 °C for five days. 

### 4.4. Polyphenol Extraction and Analysis

#### 4.4.1. Preparation of Extracts

Plant leaves of all treatments were dried at room temperature. Triplicate samples of 0.5 g dry power for each treatment were milled with 10 mL methanol solution (80%) and then ultrasonically extracted for 30 min. The mixtures were centrifuged on 4000× *g* at 4 °C for 30 min [61]. The supernatant was collected and then stored at 4 °C for evaluating contents of total phenolics and flavonoids as well as antioxidant capacity of the methanolic extracts (within 48 h).

#### 4.4.2. Determination of Total Phenolic Content (TPC)

The Folin-Ciocalteu reagent was used to determine the content of total phenolics [62]. Briefly, 3.6 mL of ddH_2_O, 0.4 mL of diluted sample extract, and 0.4 mL of Folin-Ciocalteu reagent were added sequentially to a 10-mL volumetric flask. After 5 min, 4 mL of 7% Na_2_CO_3_ solution was added. The solution was immediately made up to 10 mL with ddH_2_O and then incubated at 23 °C for 90 min. The absorbance was measured at 750 nm against a ddH_2_O blank. A standard curve was established by gallic acid (GA). Results were expressed as mg of GA equivalents on a basis of dry weight (mg GAE/g DW). All samples were analyzed in triplicate.

#### 4.4.3. Determination of Total Flavonoid Content (TFC)

The total amount of flavonoids was measured according to a modified spectrophotometric method described by Siddhuraju and Becker [63]. Prepared diluted sample (1 mL), distilled water (3 mL), and 5% NaNO_2_ (0.3 mL) were placed in a volumetric flask (10 mL) and held for 5 min. Then, 1% AlCl_3_ solution (3 mL) was added. After 6 min, 2 mL of 1 M NaOH solution was added. The reaction solution was finally diluted to 10 mL with distilled water and thoroughly mixed. Absorbance of the resulting solution was read at 510 nm against a water blank. Rutin was used to develop a standard curve. Total flavonoid content of samples was expressed as mg of rutin equivalents on a basis of dry weight (mg rutin/g DW). All samples were analyzed in triplicate.

### 4.5. Antioxidant Capacity Evaluation

#### 4.5.1. DPPH Radical Scavenging Activity

DPPH radical scavenging assay was performed following the method of Chrysargyris et al. [61]. A total of 0.02 mL leaf extract, 1.98 mL 80% methanol, and 1 mL purple DPPH solution (0.3 mM) were mixed and held for 30 min at room temperature in the dark. Absorbance of the solution at 517 nm was measured. The test was carried out in triplicate and results were presented as the inhibition percentage (%) and calculated by a given formula:

DPPH radical scavenging activity % = 100 − 100 × [(Ab_sample_ − Ab_blank_)/Ab_control_]

where Ab_sample_ is the absorbance of the test sample, Ab_blank_ is the absrbance of the blank sample and Ab_control_ is the absorbance of the control with DPPH and 80% menthol.

#### 4.5.2. ABTS^·+^ Radical Scavenging Activity

ABTS^·+^ radical scavenging assay was conducted using a modified method of Cai et al. [64]. Briefly, 2.45 mM potassium persulfate solution and 7 mM ABTS stock solution were reacted at a ratio of 0.5:1, and then they were left standing for 12–16 h in the dark at ambient temperature to generate ABTS radical cations (ABTS^·+^). The absorbance of ABTS^·+^ solution was adjusted with the addition of 80% ethanol until 0.700 ± 0.05 was read at 734 nm. Prepared 0.1 mL diluted leaf extract was reacted with 3.9 mL blue-green ABTS^·+^ solution. The absorbance at 734 nm of the resulting solution was recorded after 6 min. Trolox standard solution was employed to establish a standard calibration. The test was carried out in triplicate and results were expressed as trolox equivalent antioxidant capacity (μmol of trolox equivalent antioxidant capacity on a basis of dry weight, μmol TEAC/g DW).

### 4.6. Glandular Trichome Morphology and Density

Fresh leaf samples at the second node (from the apex) were carefully cut and any damage to the surfaces was avoided. Adaxial and abaxial leaf sides were observed with a Zeiss Stemi 2000-C stereomicroscope (SM) (Carl Zeiss Ltd., Jena, Germany) coupled with a digital camera (Canon Inc., Tokyo, Japan) for identifying glandular trichomes (GTs). A Hitachi SU8010 scanning electron microscope (SEM) (Hitachi Science System Ltd., Naka, Japan) was also used for structural details and determining glandular trichome (GT) density of both sides, and the determination was repeated fifteen times.

### 4.7. Extraction of Glandular Trichome Volatile Exudates

Extraction of GT volatile exudates was conducted following a modified version of the methods of Severson et al. [65] and Asai and Fujimoto [66]. Fresh fully extended leaves (4 g) for each treatment were collected. Three 100-mL beakers containing 50 mL methylene chloride (CH_2_Cl_2_) in each were prepared. The samples were dipped into the first beaker four times; they were kept submerged in CH_2_Cl_2_ for about 2 s/dip. Excess CH_2_Cl_2_ was allowed to run off after the fourth dip. The same procedure was performed in the other two beakers. The extracts were subjected to vacuum suction filtration (20 g anhydrous Na_2_SO_4_ was placed on the filter paper) into a clean filtering flask. Filter papers and the three beakers were washed twice with CH_2_Cl_2_ (about 150 mL). All CH_2_Cl_2_ extracts and washings were mixed in a round-bottom flask and then concentrated to 10 mL under reduced pressure at 38 °C. The concentrated solution was stored at 0 °C in the dark until analysis. Triplicate extractions were carried out for each treatment.

### 4.8. Gas Chromatography/Mass Spectrometry Analysis of GT Volatile Exudates and Compound Identification

GT volatile exudates were analyzed using a Shimadzu GC2010 gas chromatograph, interfaced with a Shimadzu QP2010 plus mass spectrometer (Shimadzu Corp., Kyoto, Japan). A RTX-5ms capillary column (30 m × 0.25 mm, 0.25 μm film thickness) (Restek, Bellefonte, PA, USA) was used. Samples of 1 μL were injected automatically and the split ratio was 20:1. Helium was the carrier gas with a flow rate of 1.3 mL/min. The temperature program was as follows: initial temperature was 50 °C, increased to 90 °C at 10 °C/min, and maintained for 5 min; increased to 160 °C at 10 °C/min and maintained for 10 min; increased to 250 °C at 10 °C/min and then maintained for 10 min. The injection temperature was 230 °C. The scan mass range was 40–800 *m*/*z*. A mass spectrometer was operated in the electron impact mode (70 eV). Compounds were identified by their unique retention indices (RI) according to the NIST05 mass spectral library. Their relative percentage was determined based on peak area normalization with no correction factors used.

### 4.9. Statistical Analysis

Data were statistically analyzed by IBM SPSS v. 19.0 (IBM SPSS Statistics Inc., Chicago, IL, USA) and presented as treatment mean ± standard deviation (SD). One-way analysis of variance (ANOVA) followed by Duncan range test was used to compare the means. For all statistical analysis, 0.05 was set as the significance level. A principal component analysis (PCA) was performed on the basis of the total peak area of detected GT volatile exudates to discriminate between different NaCl concentrations.

## 5. Conclusions

In response to increasing salinity, the dry biomass of *S. tenuifolia* decreased. The accumulation of antioxidants, including phenolics and flavonoids, was enhanced by low or moderate levels of salinity but inhibited by severe levels. Changes in the antioxidant capacity of leaf extracts were similar to those in the TPC. On both leaf surfaces, glandular trichomes (GTs) of two types were found: big peltate and small capitate. The density of total GTs on both leaf sides increased under salinities, especially under moderate to severe treatments. Salt stress significantly affected the constituents of GT volatile exudates in *S. tenuifolia*. The most abundant compound in GT volatile exudates was pulegone. The decrease, or even disappearance, of monoterpenes was induced by salt stress, while biosynthesis and accumulation of esters, especially sulfurous acid,2-ethylhexyl hexyl ester, were enhanced. Biochemical and GT density changes under salinity could reflect an adaption response of *S. tenuifolia* to this factor. In this case, salinity conditions should be avoided for the cultivation of *S. tenuifolia* due to the decrease of the marker compound, pulegone. However, mild salinity can be recommended to produce antioxidant compounds.

## Figures and Tables

**Table 1:** Total phenolic and flavonoid content and antioxidant activity of *S. tenuifolia* leaves influenced by salt stress.

NaCl Concentration (mM)	TPC (mg GAE/g DW)	TFC (mg Rutin/g DW)	DPPH (% Antioxidant Capacity)	ABTS (μmol TEAC/g DW)
0	13.24 ± 1.90 ^bc^	31.43 ± 2.64 ^b^	40.17 ± 2.41 ^c^	54.69 ± 3.26 ^b^
25	18.54 ± 0.66 ^a^	38.99 ± 0.80 ^a^	62.14 ± 2.67 ^a^	75.40 ± 5.54 ^a^
50	14.56 ± 1.32 ^b^	36.27 ± 1.01 ^a^	44.79 ± 2.31 ^b^	57.44 ± 6.73 ^b^
75	12.37 ± 0.51 ^c^	24.26 ± 1.33 ^c^	29.56 ± 1.59 ^d^	44.83 ± 1.61 ^c^
100	7.40 ± 0.32 ^d^	17.18 ± 1.72 ^d^	18.30 ± 0.70 ^e^	30.69 ± 2.21 ^d^

TPC, total phenolic content; TFC, total flavonoid content; mg GAE/g DW, mg of gallic acid equivalents on a basis of dry weight; DPPH (2,2-diphenyl-1-picrylhydrazyl); ABTS (2,2′-azinobis-(3-ethylbenzothiazoline-6-sulfonic acid)); μmol TEAC/g DW, μmol of trolox equivalent antioxidant capacity on a basis of dry weight. Values with different letters in the same column are significantly different at *p* < 0.05 (*n* = 3).

**Table 2:** Compositions of glandular trichome volatile exudates in *S. tenuifolia* and changes of their relative percentage (%) as affected by salt stress.

No.	Compounds	NaCl Concentration (mM)				
		0	25	50	75	100
1	Heptane,2,2,4,6,6-pentamethyl	2.44 ± 0.07 ^d^	7.09 ±0.19 ^b^	3.99 ± 0.13 ^c^	7.4 ± 0.18 ^b^	9.6 ± 0.21 ^a^
2	3-Heptanone,5-ethyl-4-methyl	0.21 ± 0.01 ^d^	0.79 ± 0.08 ^a^	0.34 + 0.03 ^c^	0.53 ± 0.05 ^b^	0.49 ± 0.04 ^bc^
3	D-Limonene	1.54 ± 0.01 ^a^	1.6 ± 0.01 ^a^	0.69 ± 0 ^b^	nd	nd
4	Menthone	5.48 ± 0.12 ^b^	11.74 ± 0.30 ^a^	10.81 ± 0.1 ^a^	5.67 ± 0.45 ^b^	3.1 ± 0.09 ^bc^
5	Anisic acid,tridec-2-ynyl ester	1.00 ± 0 ^d^	3.78 ± 0.16 ^bc^	1.86 ± 0.12 ^c^	5.38 ± 0.19 ^b^	13.28 ± 0.33 ^a^
6	Pulegone	78.66 ± 5.01 ^a^	63.73 ± 4.39 ^b^	63.99 ± 4.98 ^b^	48.1 ± 3.26 ^c^	31.03 ± 3.01 ^d^
7	2-Cyclohexen-1-ol,2-methyl-5-(1-methylethenyl)	3.48 ± 0.09	nd	nd	nd	nd
8	Ethanone,1-cyclopropyl-2(1-pyrrolidinyl)	nd	0.8 ± 0.00 ^b^	0.93 ± 0.01 ^a^	nd	nd
9	β-Caryophyllene	2.27 ± 0.11 ^a^	0.99 ± 0.06 ^b^	0.99 ± 0.08 ^b^	nd	nd
10	1,6-Cyclodecadiene,1-methyl-5-methylene-8-(1-methylethyl)	3.02 ± 0.14 ^a^	1.16 ± 0.09 ^b^	1.28 ± 0.07 ^b^	nd	nd
11	Glycine,N-(4-butylbenzoyl)-,butyl ester	nd	nd	nd	0.62 ± 0.02 ^a^	0.1 ± 0 ^b^
12	Ethyl propyl ketone	0.17 ± 0.02	nd	nd	nd	nd
13	Sulfurous acid,isobutyl pentyl ester	nd	0.27 ± 0.01	nd	nd	nd
14	Hexanedioic acid,bis(2-ethylhexyl) ester	nd	nd	2.29 ± 0.14 ^a^	1.44 ± 0.10 ^b^	1.76 ± 0.13 ^b^
15	2,2′-Methylenebis(6-tert-butyl-4-methyl-phenol)	0.27 ± 0.04 ^b^	nd	1.86 ± 0.19 ^a^	0.22 ± 0.23 ^b^	1.86 ± 0.20 ^a^
16	3-Hexanone,2,2-dimethyl	0.23 ± 0.01 ^e^	1.03 ± 0.08 ^b^	0.5 ± 0.02 ^d^	0.92 ± 0.07 ^bc^	2.16 ± 0.15 ^a^
17	1,4-Butanediol	0.09 ± 0.00 ^d^	0.21 ± 0.01 ^c^	0.25 ± 0.01 ^c^	0.39 ± 0.03 ^b^	0.62 ± 0.07 ^a^
18	3-Hexanone,2,5-dimethyl	0.45 ± 0.03 ^cd^	0.69 ± 0.08 ^c^	1.41 ± 0.12 ^b^	4.03 ± 0.22 ^a^	4.84 ± 0.22 ^a^
19	Sulfurous acid,2-ethylhexyl hexyl ester	nd	5.73 ± 0.71 ^bc^	7.93 ± 0.87 ^b^	24.81 ± 2.71 ^a^	26.70 ± 2.69 ^a^
	Total	99.31	99.61	99.12	99.51	98.70
**Total identified classes**						
Alkane		2.44 ± 0.36 ^d^	7.09 ± 0.58 ^b^	3.99 ± 0.41 ^c^	7.4 ± 0.61 ^b^	9.6 ± 0.78 ^a^
Ketones		1.06 ± 0.09 ^d^	3.31 ± 0.24 ^c^	3.18 ± 0.21 ^c^	5.48 ± 0.25 ^b^	9.64 ± 0.81 ^a^
Monoterpenes		89.16 ± 3.44 ^a^	77.07 ± 3.06 ^b^	75.49 ± 2.71 ^b^	53.77 ± 1.98 ^c^	34.13 ± 1.65 ^d^
Esters		1.00 ± 0.10 ^e^	9.78 ± 1.51 ^cd^	12.08 ± 1.78 ^c^	32.25 ± 2.13 ^b^	42.85 ± 3.04 ^a^
Sesquiterpene		5.29 ± 0.56 ^a^	2.15 ± 0.31 ^b^	2.27 ± 0.35 ^b^	0 ^c^	0 ^c^
Others		0.36 ± 0.04 ^d^	0.21 ± 0.02 ^d^	2.11 ± 0.16 ^ab^	0.61 ± 0.02 ^c^	2.48 ± 0.20 ^a^

Values with different letters in the same row are significantly different at *p* < 0.05 (*n* = 3). nd: not detected.

## Abbreviations

EO	essential oil
TPC	total phenolic content
TFC	total flavonoid content
NGT	non-glandular trichome
GT	glandular trichome
Pel	peltate glandular trichome
Cap	capitate glandular trichomes
SEM	scanning electron microscope
